# Radiomic Signature Based on Dynamic Contrast-Enhanced MRI for Evaluation of Axillary Lymph Node Metastasis in Breast Cancer

**DOI:** 10.1155/2022/1507125

**Published:** 2022-08-17

**Authors:** Yanqiu Tang, Lin Chen, Yating Qiao, Weifeng Li, Rong Deng, Mengdi Liang

**Affiliations:** ^1^Department of Breast Surgery, The First Affiliated Hospital with Nanjing Medical University, Nanjing 210029, China; ^2^Department of General Surgery, The Affiliated Cancer Hospital of Nanjing Medical University & Jiangsu Cancer Hospital & Jiangsu Institute of Cancer Research, Nanjing 210009, China; ^3^Department of Gastrointestinal Surgery, Affiliated Hospital of Hebei University, Baoding, China; ^4^School of Electronic Science and Engineering, Nanjing University, Nanjing 210046, China

## Abstract

**Background:**

To construct and validate a radiomic-based model for estimating axillary lymph node (ALN) metastasis in patients with breast cancer by dynamic contrast-enhanced magnetic resonance imaging (DCE-MRI).

**Methods:**

In this retrospective study, a radiomic-based model was established in a training cohort of 236 patients with breast cancer. Radiomic features were extracted from breast DCE-MRI scans. A method named the least absolute shrinkage and selection operator (LASSO) was applied to select radiomic features based on highly reproducible features. A radiomic signature was built by a support vector machine (SVM). Multivariate logistic regression analysis was adopted to establish a clinical characteristic-based model. The performance of models was analysed through discrimination ability and clinical benefits.

**Results:**

The radiomic signature comprised 6 features related to ALN metastasis and showed significant differences between the patients with ALN metastasis and without ALN metastasis (*P* < 0.001). The area under the curve (AUC) of the radiomic model was 0.990 and 0.858, respectively, in the training and validation sets. The clinical feature-based model, including MRI-reported status and palpability, performed slightly worse, with an AUC of 0.784 in the training cohort and 0.789 in the validation cohort. The radiomic signature was confirmed to provide more clinical benefits by decision curve analysis.

**Conclusions:**

The radiomic-based model developed in this study can successfully diagnose the status of lymph nodes in patients with breast cancer, which may reduce unnecessary invasive clinical operations.

## 1. Introduction

Breast cancer has severely threatened women's physical health and quality of life. It accounts for the highest incidence of malignancy and the second leading cause of tumour-related deaths in females [1]. The 5-year survival rate is up to 99% for patients with localized breast cancer, while the rate of patients with lymph node metastasis descends to 89% [2].

The identification of axillary lymph node (ALN) is crucial for therapy of patients with breast cancer, which determines whether postoperative chemical or radiation therapy is needed [3]. Sentinel lymph node dissection (SLND) is recommended to predict the status of nonpalpable axillary lymph nodes [4]. Axillary lymph node dissection (ALND) or ultrasound-guided fine-needle aspiration (US-FNA) is clinically operated in patients with palpable axillary lymph nodes [5]. However, both ALND and SLND are invasive, potentially resulting in impaired shoulder range of motion, limber edema, numbness, and pain syndromes [6, 7]. Therefore, it would be beneficial if there is a reliable noninvasive evaluation of ALN metastasis.

Radiomics has attracted great attention as a potential way to preoperatively detect breast cancer and assess ALN status. Quantitative imaging features extracted from magnetic resonance imaging (MRI), combined with other clinical information, can be used to support clinical decisions [8]. In the study by Chai et al. [9], dynamic contrast-enhanced magnetic resonance imaging (DCE-MRI) showed the best performance to predict ALN metastasis among multiparametric MR sequences.

There have been several studies evaluating SLN status by radiomic analysis of DCE-MRI combined with clinicopathologic features. However, the use of ALND shows no superior survival compared with SLND alone for patients with T1 or T2 breast cancer if there are only one or two positive SLNs [10, 11]. Thus, patients with positive SLNs may undergo unnecessary ALND due to the lack of evidence for ALN metastasis [12]. The purpose of this study is to predict ALN metastasis by developing and validating a radiomics-based model.

## 2. Methods

### 2.1. Patients

**Figure 1:**
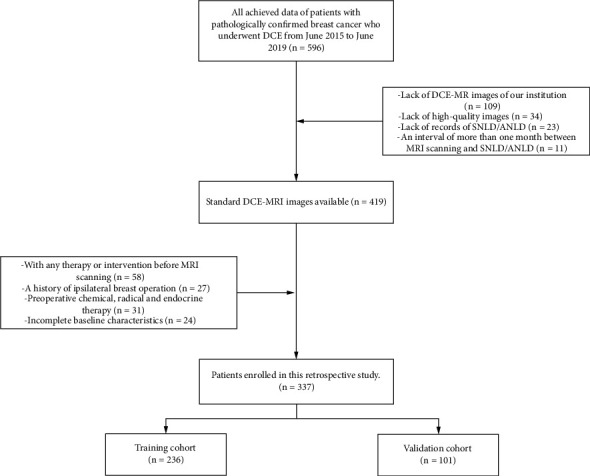
Patient selection flow chart. In all, 337 breast cancer patients with high-quality DCE-MRI scans were included in this study.

573 patients with histologically confirmed breast cancer who received DCE-MRI and SLND/ALND from June 2015 to June 2019 were retrospectively reviewed. The exclusion criteria were (1) lack of DCE-MR images at The Affiliated Cancer Hospital of Nanjing Medical University (*n* = 109). (2) lack of high-quality images (*n* = 34), (3) an interval of more than one month between MRI scanning and SNLD/ANLD (*n* = 11), (4) with any therapy or intervention before MRI scanning (e.g., a history of ipsilateral breast operation and chemical, radical, and endocrine therapy) (*n* = 58), and (5) incomplete baseline characteristics (*n* = 24). A total of 337 patients were enrolled in this study (Figure 1). Based on the date of surgery, 236 patients treated from June 2015 to December 2017 were allocated to the training cohort and 101 patients from January 2018 to June 2019 were for the validation cohort.

Clinical characteristics and the data of baseline MR imaging were collected from medical records. The clinical data included age, immunophenotype (according to progesterone receptor (PR) status, estrogen receptor (ER) status, human epidermal growth factor receptor 2 (HER-2) status, and Ki67 proliferation index), histological type (invasive ductal carcinoma, invasive lobular carcinoma, and other types), and ALN palpability. The serum tumour markers (cancer antigen 15-3 (CA15-3), cancer antigen 125 (CA125), and carcinoembryonic antigen (CEA)) were considered due to the diagnostic value in metastatic breast cancer [13].

### 2.2. SLND/ALND and Pathological Assessment

SLND was performed for all participants by the dye method within one week after MRI examination. Negative indications of ALND were defined as isolated tumour cells and micrometastasis in the SN (isolated tumour cells: <0.2 mm or <200 tumour cells; micrometastasis: tumour diameter > 0.2 mm, ≤2 mm, or <200 tumour cells). Macrometastasis (tumour diameter > 2 mm) was considered a positive indication of axillary clearance [14].

Histological types of breast cancer were analysed by two pathologists with over 5-year experience in consensus. Each pathologist was blinded to the clinical situation. Immunophenotypes were based on expression levels of ER, PR, HER2, and Ki67: luminal A (ER+ and/or PR+, HER2-, and Ki67-low), luminal B (ER+ and/or PR+, HER2+; ER+ and/or PR+, HER2-, and Ki67-high), HER2 positive (ER-, PR-, and HER2+), and triple-negative (ER-, PR-, and HER2-) [15].

### 2.3. MR Image Acquisition and Radiologic Evaluation

DCE-MRI scanning was performed with a 3 T MRI system (SIEMENS) and 8-channel breast coils in a prone position. The contrast enhancement agent, gadopentetate dimeglumine penta-acetic acid (Gd-DPTA), was intravenously injected at a dose of 0.1 mmol/kg and a rate of 3.0 ml/s. A total of five phases, one precontrast and four postcontrast phases, were obtained with a sagittal VIBRANT multiphase sequence: repetition time (TR) = 4.46-7.80 msec, echo time (TE) = 1.54-4.20 msec, flip angle = 10°, field of view (FOV) = 36∗36 cm^2^, matrix = 256∗256, and slice thickness without a gap = 2 mm.

**Figure 2:**
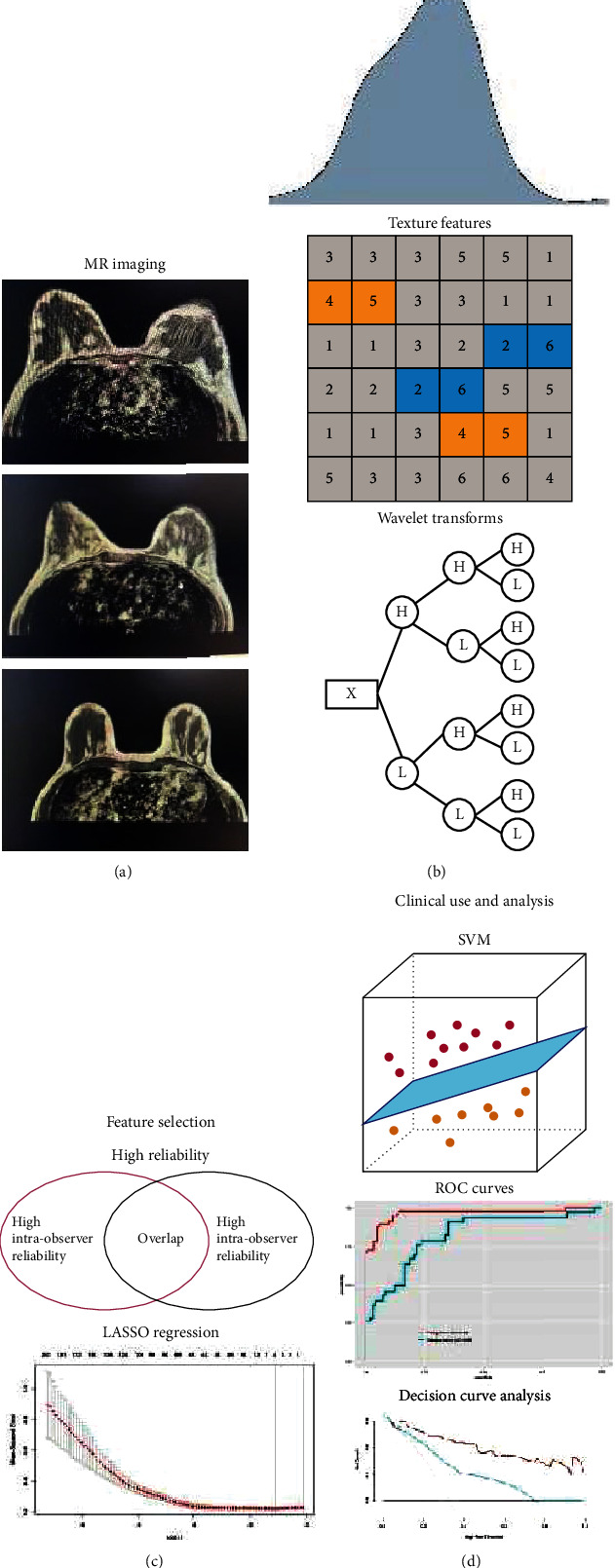
The workflow of necessary steps in this study. (a) ROI was manually delineated on the DCE-MR images. (b) Radiomic features were extracted, including the first-order statistics, textural features, and wavelet transforms. (c) Radiomic features highly related to LN metastasis were selected to construct a radiomic signature. (d) The radiomic model was constructed by SVM, and the performance of models was evaluated by ROC and DCA curves.

The workflow is depicted in Figure 2. Two radiologists, one with 8 years of breast cancer MRI experience and the other with 10 years, evaluated the following traits of all images with blindness to the clinical and pathological details: (1) tumour size: defined by the maximum transverse diameter of the largest lesion; (2) multifocality: defined by more than one lesion; and (3) LN status: defined by shape, fatty hilum, cortical thickness, and thickening pattern [16]. Consensus would be reached through discussion if the two radiologists disagreed with the LN status.

### 2.4. Region-of-Interest Segmentation and Radiomic Feature Extraction

The signal intensity of tumour lesions in different phases was calculated with GE Advanced Workstation ADW4.4. In the images with the strongest enhanced phase, region of interests were manually segmented along the 4 mm dilated tumour contour by a 3D slicer (version 4.4.0), which contains both intra- and peritumoural data.

Firstly, to assess inter- and intraobserver reliability, the ROIs were performed by two experienced radiologists (one with 10-year experience and the other with 16-year experience). Reader 1 repeated the segmentation twice a week, and reader 2 independently extracted ROIs to, respectively, calculate intra- and interobserver reproductivity with intraclass correlation coefficient [17] (ICC). Radiomic features with both intra- and interobserver ICC greater than 0.8 were subsequently analysed in this study. Secondly, the least absolute shrinkage and selection operator [18] (LASSO), with penalty parameter tuning conducted by 10-fold cross-validation, was applied to select features of ALN status with nonzero coefficients in the training cohort.

### 2.5. Establishment, Performance, and Validation of a Radiomic Model

The support vector machine (SVM) is a kind of supervised model for regression analysis with robust prediction ability. Based on selected radiomic features, SVM was applied to generate a radiomic signature, using “e1071” package (https://CRAN.R-project.org/package=e1071) on R software (version 3.6.1, http://www.r-project.org).

To assess the association between ALN metastasis and clinical features, the features with significant differences (*P* < 0.05) between the training and validation cohorts were selected for further analysis. Next, multivariable logistic regression was applied to build the clinical model in the training cohort. The cutoff value of each independent risk factor was evaluated by receiver operating characteristic (ROC) analysis with the maximum Youden index.

This study established three models to predict ALN metastasis using logistic regression, including the radiomic signature alone, clinical factors alone, and the model combining the radiomic signature and clinical risk factors. The discrimination performance of each model was determined by ROC analysis and area under the curve (AUC). The Delong test was used to compare each model with the AUC value. The performance of models was then tested in the independent validation cohort with the formula from the training cohort.

### 2.6. Statistical Analysis

For categorical variables, the chi-squared test or Fisher exact test was performed to analyse the equality of variances between the training and validation cohorts, and the Student *t*-test or Mann–Whitney *U* test was used to compare continuous variables. Decision curve analysis (DCA) was applied in the validation cohort to assess the benefit of each model at different threshold probabilities. A two-sided *P* value less than 0.05 was considered statistical significance.

## 3. Results

### 3.1. Patient Characteristics

As summarized in Table 1, there were no statistical differences in clinical and radiological characteristics between the training and validation cohorts. The rates of LN metastasis were, respectively, 33.1% (78 of 236) and 35.6% (36 of 101) in the training and validation cohorts, whereas no difference was found between the two cohorts (*χ*^2^, *P* = 0.64). The overall discrimination accuracy of MRI report of LN status was 63%, with a sensitivity of 48.7% (38 of 78), a specificity of 66.4% (105 of 158), a positive value of 76.0% (38 of 50), and a negative predictive value of 56.4% (105 of 186). Statistical differences were found between non-LN metastasis and LN metastasis in multifocality, LN palpability, and MRI-reported LN status (Table 2).

### 3.2. Radiomic Signature

In total, 841 features (13 shape features, 18 first-order features, 74 textural features, and 736 wavelet-based features) were automatically extracted from each ROI with the opensource Pyradiomics package (http://www.radiomics.io/pyradiomics.html). Details about the extracted features are shown in the supplement material (Table S1).

**Figure 3:**
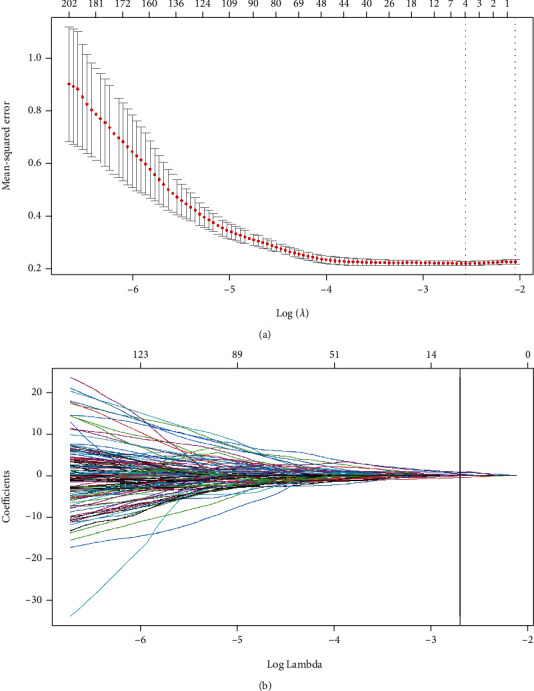
Radiomic feature selection by the least absolute shrinkage and selection operator (LASSO) regression. (a) LASSO coefficient profiles of the 841 selected features. (b) Optimal *λ* value was determined by the LASSO model using 10-fold cross-validation via minimum criteria. The mean-squared error was plotted versus log(*λ*). Dotted vertical lines were drawn at the optimal values by using the minimum criteria and the 1 standard error of the minimum criteria. The optimal *λ* value of 0.067 was chosen.

Of 841 extracted features, 332 LN-related features were selected for the following analysis, including 11 shape features, 9 first-order features, 37 textural features, and 275 wavelet-based features. Five LN status-related features with nonzero coefficients in the LASSO regression model were selected based on the training cohort, including two shape features, one textural feature, and two wavelet-transformed features (Figure 3). The five features are shown in Table 3.

**Figure 4:**
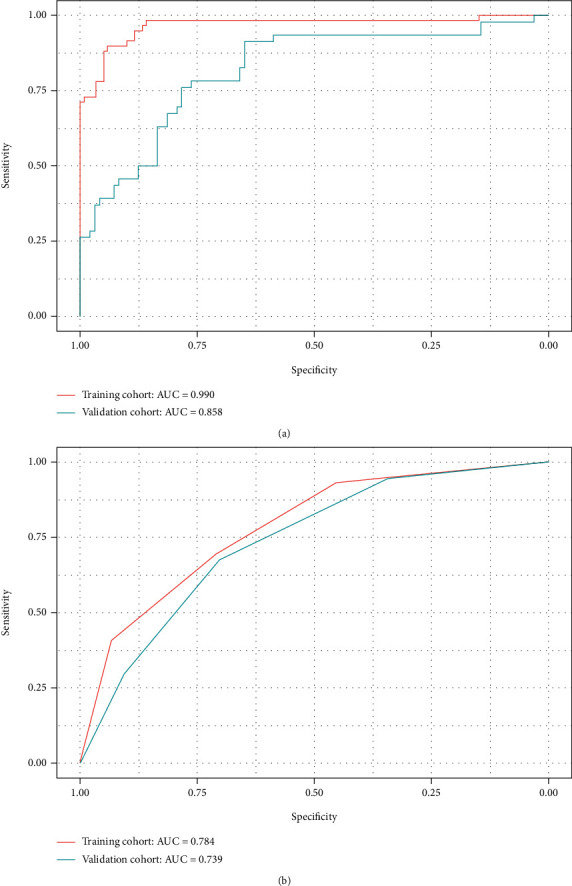
ROC curves using radiomic features (a) and clinical features (b) for the training and validation sets.

The SVM algorithm was applied to construct a radiomic signature. A difference in the decision values was observed between patients with and those without LN metastasis in the training cohort (mean, -0.581 vs. 0.737, *P* < 0.001) and also obtained in the validation cohort (mean, 0.129 vs. 0.470, *P* < 0.001). As is shown in Figure 4, the radiomic signature displayed a favourable discriminatory ability with an AUC of 0.990 (95% confidence interval (CI): 0.990-1) in the training cohort and 0.858 (95% CI: 0.834-0.950) in the validation cohort. The optimal cutoff value of 0.8567 for the radiomic signature was calculated at the point of the maximum Youden index from the entire cohort. The radiomic model performs well in the training cohort, whose sensitivity and specificity were 100% and 94%, respectively. In the validation cohort, the sensitivity was as high as 87% and the specificity was 78%. The accuracies were 96% and 81% in the training and validation cohorts, respectively. The calibration curve of the radiomic signature yielded great agreement between the predicted and actual metastases in the training cohort.

### 3.3. LN Status-Related Clinical Factors

As shown in Table 1, histological type, multifocality, MRI-reported LN status, and LN palpability were significantly related to LN metastasis (*P* < 0.001, chi-squared test).  Table 4 displays the odds ratios of the above clinical factors. The odds ratios of MRI-reported status and LN palpability were statistically significant, respectively, 5.28 (95% CI: 2.52-11.79) and 7.35 (95% CI: 3.48-16.57). Then, the clinical prediction model was built by multivariable logistic regression based on MRI-reported status and LN palpability. The model displayed an AUC of 0.784 (95% CI: 0.716-0.851) in the training cohort, and the sensitivity, specificity, and accuracy were, respectively, 69%, 71%, and 70%. The performance in the validation cohort was similar, with an AUC of 0.739 (95% CI: 0.644-0.833), a sensitivity of 68%, a specificity of 70%, and an accuracy of 69%. The optimal cutoff value of the clinical model was -0.671, determined from the whole cohort. Compared with the radiomic signature, the clinical model yielded poorer results in the training and validation sets.

### 3.4. Combining Radiomic Signature and Clinical Factors

The discriminatory ability of the combined model was poorer than that of the radiomic signature, with an AUC of 0.987 (95% CI: 0.9743-1; *P* = 0.877) in the training cohort and of 0.826 (95% CI: 0.742-0.909; *P* = 0.11). The sensitivity, specificity, and accuracy of the third model were, respectively, 96%, 93%, and 95% in the training cohort and 77%, 81%, and 78% in the validation cohort.

### 3.5. Clinical Use

**Figure 5:**
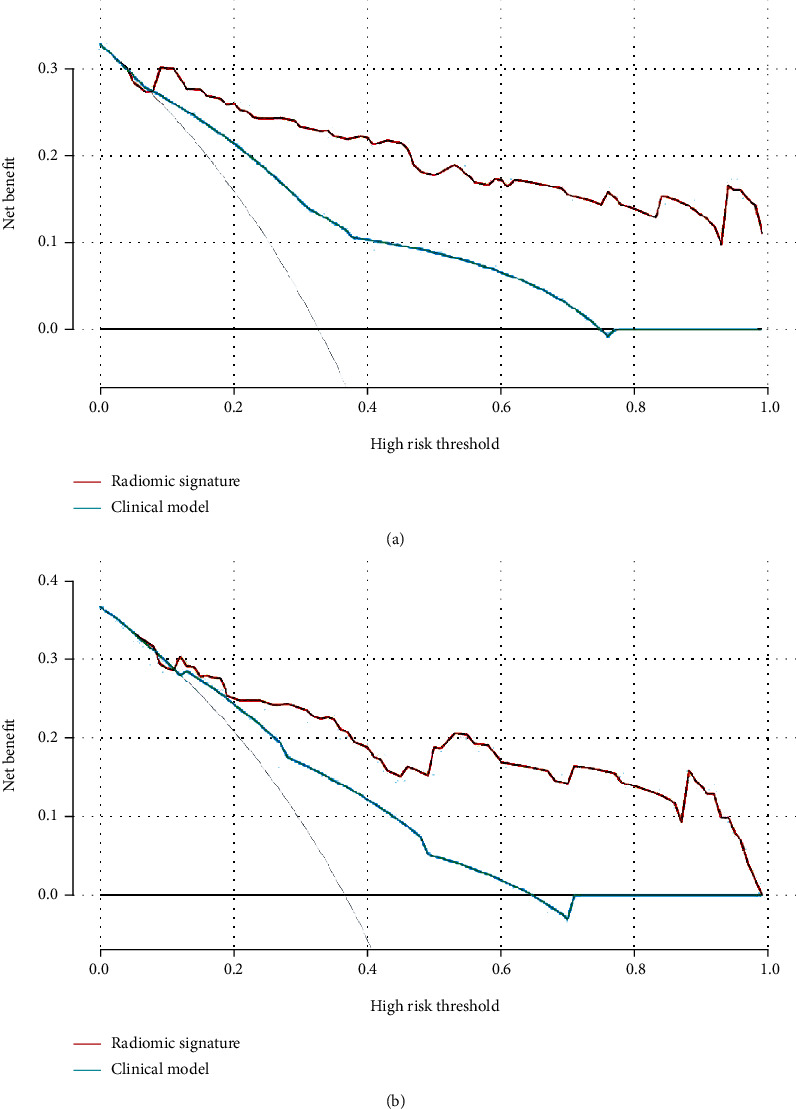
Decision curve analysis for each model in the training (a) and validation (b) cohorts. The *Y*-axis measures the net benefit, calculated by the true-positive findings and false-positive findings. Across the threshold probability, the application of radiomic signature to predict LN status provides more benefits than clinical features.

As summarized in Table 5, the radiomic signature had the best discriminatory ability in the training and validation cohorts. In the training cohort, the AUC value of the radiomic signature was significantly higher than that of the clinical model (AUC: 0.784; CI: 0.716-0.851), MRI-reported metastasis alone (AUC: 0.661; CI: 0.586-0.735; *P* < 0.001), and palpability (AUC: 0.703; CI: 0.631-0.775; *P* < 0.001). In the validation cohort, the radiomic signature displayed the best results, compared with the clinical model (AUC: 0.739; CI: 0.644-0.833; *P* = 0.020), the MRI-reported metastasis (AUC: 0.557; CI: 0.456-0.659; *P* < 0.001), and palpability (AUC: 0.689; CI: 0.594-0.784; *P* = 0.002). Though the AUC of the radiomic signature was slightly higher than the combined model, the difference showed no statistical significance.  Figure 5 presents the decision curve analysis for the clinical prediction model and the radiomic signature. The radiomic signature indicates more benefit to predict LN metastasis, with the threshold probabilities of more than 10%.

## 4. Discussion

This study constructed and validated a radiomics-based model and a clinical model to predict LN metastasis in patients with breast cancer. Six stable radiomic features effectively identify patients as LN metastasis or non-LN metastasis. Compared with the clinical model consisting of the MRI-reported LN status and LN palpability, the radiomic model performed much better with an AUC of 0.858 in the validation cohort.

Radiomics is termed as extracting quantitative features that convert images into mineable data, and analyse these data to improve diagnosis, prediction power, and much other decision support. MRI-based radiomic analysis can provide an efficient method to estimate the existence of ALN metastasis and probably change the clinical routine in the future. In this study, we constructed a radiomic model based on images extracted from DCE-MRI to find the LN metastasis, and the results of the model were satisfactory. The AUC, sensitivity, and specificity were 0.858, 87%, and 78%, respectively, in the validation cohort. While MRI has been the main noninvasive method to assess LNs, its sensitivity and specificity were merely 68% and 70%.

Like other research, this study found that the clinical characteristics of patients with breast cancer were related to LN status, such as multifocality, palpability of LNs, and MRI-reported LN status. Tan et al. selected four factors, including age, HER2 status, size of tumour, and vascular thrombus accompanied or not, into the clinical model to predict SLN metastasis [19]. The accuracy of the model relying on clinicopathological features was merely 70.26%, much lower than the radiomic signatures, and the damage must happen if histological information is needed for the model.

A radiomic signature, based on ROI extracted from DCE-MR images, was constructed in this study to evaluate the ALN metastasis. Dong et al. predicted SLN metastasis based on radiomics of diffusion-weighted (DWI) and T2-weighted fat-suppression MRI (T2-FS). The AUC of radiomic model-based DWI and T2-FS were, respectively, 0.77 and 0.79 in the validation set, slightly lower than ours. To date, there have been some articles published combining the radiomic signature and clinicopathological features. For instance, Han et al. evaluated the ALN with the radiomic signature and clinical characteristics including palpability of LN and MRI-reported LN status, achieving an AUC of 0.78 [20]. Similar clinical features associated with LN metastasis were found to predict the LN status in our study, but the radiomic-based model combining the clinical features was slightly worse than the model using the radiomic signature alone. One of the reasons was that the accuracy of the clinical information is much dependent on the experience of doctors.

There were several limitations in this study. First, the retrospective analysis had inherent bias influencing the outcomes. More independent samples from different centres will be needed to validate the results. Second, the images to extract radiomic features are the primary tumour instead of LNs. The MR images with LNs, however, are only a small part of all samples. Third, the ROI is circled manually. Although the intra- and interobserver ICC were more than 0.8, some studies demonstrated that automated or semiautomated methods show higher accuracy and stability [21, 22]. Fourth, many scholars have applied radiogenomics to cancer research [23–25]. This study ignored the genomic data due to economic limitations.

In conclusion, the radiomic model is a promising noninvasive method to predict LN metastasis for breast cancer. Further study with a larger sample size is needed to achieve the application.

### 4.1. Clinical Practice Points

Breast cancer patients with positive SLNs are advised to undergo ALND to confirm the ALN status which is important for treatment strategy, while some results of ALND are negative. We hope to find a convenient and noninvasive method for patients with breast cancer to assess ALN status. MRI has been extensively applied to the diagnosis of breast cancer, and it is easy for patients to obtain MR images. The MRI-based radiomic model performs well in evaluating the ALN metastasis and may reduce unnecessary lesions.

## Figures and Tables

**Table 1:** Main characteristics of patients in the training and validation cohorts.

Characteristics	Training (*n* = 236)	Validation (*n* = 101)	*P*
Age^∗^	53.21 ± 9.72	52.7 ± 10.13	0.67
Immunophenotype			0.415
Luminal A	76 (32.2)	38 (37.6)	
Luminal B	126 (53.4)	46 (45.5)	
HER2	11 (4.7)	8 (7.9)	
Triple-negative	23 (9.7)	9 (8.9)	
Histological type			0.547
Invasive carcinoma	189 (80.1)	87 (86.1)	
Precursor lesions	43 (18.2)	14 (13.9)	
Tumour markers^∗^			
CEA	0.83 ± 0.31	0.76 ± 0.42	0.09
CA15-3	12.32 ± 5.41	11.71 ± 5.53	0.34
CA125	20.61 ± 8.57	21.13 ± 7.49	0.59
Tumour size	18.44 ± 8.34	19.52 ± 9.12	0.29
Multifocality			0.56
Yes	21 (8.9)	11 (10.9)	
No	215 (91.1)	90 (89.1)	
LN palpability			0.88
Yes	29 (12.3)	13 (12.9)	
No	207 (87.1)	88 (87.1)	
MRI-reported LN status			0.48
Positive	50 (21.2)	18 (17.8)	
Negative	186 (78.8)	83 (82.2)	
LN metastasis			0.68
Yes	78 (33.1)	36 (35.6)	
No	158 (66.9)	65 (64.4)	

Note. Data are numbers of patients, with percentages in parentheses. CEA: carcinoembryonic antigen; CA15-3: cancer antigen 15-3; CA125: cancer antigen 125. ^∗^Data are presented as means ± standard deviations.

**Table 2:** Main characteristics of patients with and without LN metastasis.

Characteristics	LN metastasis (*n* = 114)	Non-LN metastasis (*n* = 223)	*P*
Age^∗^	52.13 ± 9.18	52.75 ± 8.93	0.54
Immunophenotype			0.29
Luminal A	41 (40.0)	73 (32.7)	
Luminal B	51 (44.7)	121 (54.2)	
HER2	9 (7.9)	10 (4.5)	
Triple-negative	13 (11.4)	19 (8.5)	
Histological type			<0.001
Invasive carcinoma	111 (97.4)	169 (75.8)	
Precursor lesions	3 (2.6)	54 (24.2)	
Tumour markers^∗^			
CEA	0.77 ± 0.45	0.82 ± 0.31	0.26
CA15-3	11.69 ± 5.61	12.37 ± 5.32	0.27
CA125	20.53 ± 7.86	20.88 ± 8.41	0.71
Tumour size	19.82 ± 7.58	18.22 ± 9.42	0.12
Multifocality			<0.001
Yes	22 (19.3)	10 (4.5)	
No	92 (80.7)	213 (95.5)	
LN palpability			<0.001
Yes	34 (29.8)	8 (3.6)	
No	80 (80.2)	215 (96.4)	
MRI-reported LN status			<0.001
Positive	46 (40.4)	22 (9.9)	
Negative	68 (59.4)	201 (90.1)	
LN metastasis			
Yes	—	—	
No	—	—	

Note. Data are numbers of patients, with percentages in parentheses. CEA: carcinoembryonic antigen; CA15-3: cancer antigen 15-3; CA125: cancer antigen 125. ^∗^Data are presented as means ± standard deviations.

**Table 3:** LN status-related radiomic features.

Types	Features
Shape (*n* = 2)	Maximum 3D diameter, minor axis
Texture features (*n* = 1)	Original GLDM gray level variance
Wavelet transforms (*n* = 2)	LHL GLSZM gray level nonuniformity, HHL glcm difference entropy

Note. GLDM: gray level difference matrix; GLSZM: gray level size zone matrix; H: high; L: low.

**Table 4:** Clinical risk factors for axillary lymph node metastasis.

	*b* coefficient	Odds ratio	*P* value
Histological type	0.26	1.3 (0.23-11.45)	0.7864
Multifocality	0.12	1.13 (0.33-3.67)	0.8444
MRI-reported status	1.66	5.28 (2.52-11.79)	<0.001
LN palpability	2	7.35 (3.48-16.57)	<0.001

Note. *b* coefficient was from multivariable logistic regression. Clinical factors found to be significantly related to the LN metastasis entered into the clinical model.

**Table 5:** Performances of all methods for predicting LN metastasis.

	Training	Validation	Training vs. validation
	AUROC	AUROC	Delong test
Radiomic signature	0.990 (0.990, 1)	0.858 (0.834, 0.950)	0.004
Clinical model	0.784 (0.716, 0.851)	0.739 (0.644, 0.833)	0.4439
Combined model	0.987 (0.9743-1)	0.826 (0.742-0.909)	<0.001
MRI-reported status	0.661 (0.586-0.735)	0.557 (0.456-0.659)	0.1083
LN palpability	0.703 (0.631-0.775)	0.689 (0.594-0.784)	0.8253
Comparison of AUCROC			
Radiomic signature vs. clinical model	<0.001	0.02	
Radiomic signature vs. MRI-reported status	<0.001	<0.001	
Radiomic signature vs. LN palpability	<0.001	0.002	
Radiomic signature vs. combined model	0.877	0.11	
Clinical model vs. MRI-reported status	0.005	0.006	
Clinical model vs. LN palpability	<0.001	0.028	
MRI-reported status vs. palpability	0.462	0.103	

Note. 95% confidence intervals were shown in parentheses.

## Data Availability

The data used to support the findings of this study are available from the corresponding author upon request.

## Supplementary Materials

Supplementary Materials The supplementary material contains a table showing all 841 features extracted from ROI. Table S1: list of all extracted features.
