# Supplementary material for: Radiomic Signature Based on Dynamic Contrast-Enhanced MRI for Evaluation of Axillary Lymph Node Metastasis in Breast Cancer
Source: Comput Math Methods Med. 2022 Aug 17;2022:1507125. doi: 10.1155/2022/1507125 (PMC9402328; doi:10.1155/2022/1507125)
Supplement: Supplementary Materials — The supplementary material contains a table showing all 841 features extracted from ROI. Table S1: list of all extracted features. [file 1507125.f1.docx]

Table S1. List of All Extracted Features

| **Types** | **Features** |
| --- | --- |
| **Shape**  **(n=13)** | Maximum3DDiameter, Maximum2DDiameterSlice, Sphericity, MinorAxis, Elongation, SurfaceVolumeRatio, Volume, MajorAxis, SurfaceArea, Flatness, LeastAxis, Maximum2DDiameterColumn, Maximum2DDiameterRow |
| **First-order statistics**  **(n=18)** | InterquartileRange,Skewness, Uniformity, Median, Energy, RobustMeanAbsoluteDeviation, MeanAbsoluteDeviation, TotalEnergy, Maximum, RootMeanSquared, 90Percentile, Minimum, Entropy, Range, Variance, 10Percentile, Kurtosis, Mean |
| **Textural features(n=74)**  **GLDM**  **(n=14)** | Maximum2DDiameterColumn, Maximum2DDiameterRow, GrayLevelVariance, HighGrayLevelEmphasis, DependenceEntropy, DependenceNonUniformity, GrayLevelNonUniformity SmallDependenceEmphasis, SmallDependenceHighGrayLevelEmphasis, DependenceNonUniformityNormalized, LargeDependenceEmphasis, LargeDependenceLowGrayLevelEmphasis, DependenceVariance, LargeDependenceHighGrayLevelEmphasis, SmallDependenceLowGrayLevelEmphasis, LowGrayLevelEmphasis, |
| **GLCM**  **(n=23)** | JointAverage, SumAverage, JointEntropy, ClusterShade, MaximumProbability, Idmn, JointEnergy, Contrast, DifferenceEntropy, InverseVariance, DifferenceVariance, Idn, Idm, Correlation, Autocorrelation, SumEntropy, SumSquares, ClusterProminence, Imc2, Imc1, DifferenceAverage, Id, ClusterTendency |
| **GLRLM**  **(n=16)** | ShortRunLowGrayLevelEmphasis, GrayLevelVariance, LowGrayLevelRunEmphasis, GrayLevelNonUniformityNormalized, RunVariance, GrayLevelNonUniformity, LongRunEmphasis, ShortRunHighGrayLevelEmphasis, RunLengthNonUniformity, ShortRunEmphasis, LongRunHighGrayLevelEmphasis, RunPercentage, LongRunLowGrayLevelEmphasis, RunEntropy, HighGrayLevelRunEmphasis, RunLengthNonUniformityNormalized |
| **GLSZM**  **(n=16)** | GrayLevelVariance, ZoneVariance, GrayLevelNonUniformityNormalized, SizeZoneNonUniformityNormalized, SizeZoneNonUniformity, GrayLevelNonUniformity, LargeAreaEmphasis, SmallAreaHighGrayLevelEmphasis, ZonePercentage, LargeAreaLowGrayLevelEmphasis, LargeAreaHighGrayLevelEmphasis, HighGrayLevelZoneEmphasis, SmallAreaEmphasis, LowGrayLevelZoneEmphasis, ZoneEntropy, SmallAreaLowGrayLevelEmphasis |
| **NGTDM**  **(n=5)** | Coarseness, Complexity, Strength, Contrast, Busyness |
| **Wavelet transforms**  **(n=736)** | First-order and textural features are transformed by Wavelet (wavelet-LHL, wavelet-LHH, wavelet-HLL, wavelet-LLH, wavelet-HLH, wavelet-HHH, wavelet-LLL) |

Note. GLDM, Gray Level Difference Matrix; GLRLM, Gray Level Run Length Matrix; GLSZM, Gray Level Size Zone Matrix; NGTDM, Neighborhood Gray-Tone Difference Matrix; H, high; L, Low.
